# Tomato rot by *Rhizopus microsporus* alters native fungal community composition and secondary metabolite production

**DOI:** 10.3389/fmicb.2025.1508519

**Published:** 2025-01-30

**Authors:** Mmanoko Napo, Alicia Kock, Kazeem A. Alayande, Michael Sulyok, Chibundu N. Ezekiel, Jessie Uehling, Teresa E. Pawlowska, Rasheed A. Adeleke

**Affiliations:** ^1^Unit of Environmental Sciences and Management, North-West University, Potchefstroom, South Africa; ^2^Department of Agrobiotechnology, Institute of Bioanalytics and Agro-Metabolomics, University of Natural Resources and Life Sciences Vienna, Tulln, Austria; ^3^Feed and Food Quality, Safety and Innovation GmbH, Tulln, Austria; ^4^Department of Botany and Plant Pathology, Oregon State University, Corvallis, OR, United States; ^5^School of Integrative Plant Science, Plant Pathology & Plant-Microbe Biology, Cornell University, Ithaca, NY, United States

**Keywords:** fungal community shift, *Mycetohabitans endofungorum*, secondary metabolites, *Rhizopus microsporus*, spoilage

## Abstract

*Rhizopus* rot is considered one of the most common diseases influencing global production and yield of horticulture commodities. However, the factors contributing to this pattern of prevalence are uncertain. Here, we focused on *R. microsporus*, which is known to rely on its endosymbiotic bacterium, *Mycetohabitans*, to produce toxins that interfere with plant development and inhibit the growth of other fungi. We assessed the impact of the symbiotic *R. microsporus* harboring its endosymbiont as well as the fungus cured of it on: (1) the magnitude of spoilage in tomato fruits, as evaluated by Koch's postulate for pathogenicity, (2) the shifts in native communities of endophytic fungi inhabiting these fruits, as examined by ITS rRNA gene metabarcoding and (3) secondary metabolites generated by these communities, as analyzed using multi-analyte LC-MS/MS. The pathogenicity test showed that the symbiotic endobacterium-containing *R. microsporus* W2-50 was able to cause tomato fruit spoilage. This was accompanied by decreased relative abundance of *Alternaria* spp. and an increase in the relative abundance of *Penicillium* spp. that may have facilitated the observed spoilage. In conclusion, symbiotic W2-50 appeared to facilitate fruit spoilage, possibly through successful colonization or toxin production by its endosymbiont.

## Introduction

The genus *Rhizopus* comprises cosmopolitan filamentous fungi found in the terrestrial environment, either as soil saprotrophs, pathogens of plants or opportunistic pathogens of animals (Jennessen et al., 2005; Dolatabadi et al., 2014). *Rhizopus* spp. are not only infamous for causing mucormycosis in animals and humans but also for spoilage of various economically important agricultural commodities (Kwon and Lee, 2006; Baggio et al., 2016; Pervez et al., 2023; Liu et al., 2024). *Rhizopus* rot is caused by several species of the genus *Rhizopus* such as *R. stolonifer* (Kwon et al., 2001), *R. oryzae* (Kwon et al., 2012; Khokhar et al., 2019; Li et al., 2023)*, R. arrhizus* (Yang et al., 2020; Bhuiyan et al., 2022), and *R. microsporus* (Kabiru and Yusuf, 2024). Spoilage induced by *R. microsporus* in agricultural commodities was reported in groundnuts (Steyn et al., 1983), sunflowers, *Helianthus annuus* L. (Swart, 1988), and apples, *Malus domestica* var. Golden delicious (Pervez et al., 2023). Seedlings of rice (*Oryza sativa*) infected with *Rhizopus microsporus* experienced rice seedling blight (Iwasaki et al., 1984). Generally, *Rhizopus* rot is characterized by soft, watery, slightly sunken lesions accompanied by progressive growth of whitish-gray mycelia with black sporangia (Baggio et al., 2016). Favorable growth conditions for the pathogen eventually lead to the overall decay of infected commodities, causing significant agricultural and economic losses. *Rhizopus* spp. have been previously discovered to harbor toxin-producing endobacteria that facilitate fungal virulence in plants and animals (Partida-Martinez and Hertweck, 2005; Partida-Martinez et al., 2007a; Richter et al., 2022).

Toxic rhizonin and rhizoxin metabolites of endobacterial origin have been discovered in species of the *R. microsporus* group isolated from Mozambican groundnuts, CBS 112285 (Steyn et al., 1983; Jennessen et al., 2005; Partida-Martinez et al., 2007a) and diseased rice seedlings, Rh-2 or ATCC 62417 (Iwasaki et al., 1984; Partida-Martinez and Hertweck, 2005), respectively. The 16S rRNA gene phylogenies from both plant-pathogenic fungi identified bacterial symbionts of the genus *Mycetohabitans* namely, *M. rhizoxinica* and *M. endofungorum* as the true producers of these toxins (Partida-Martinez and Hertweck, 2005; Partida-Martinez et al., 2007a,b).

Endobacterial symbionts are known to provide numerous advantages to their fungal hosts. They aid in fending off fungivorous micro-predators (Richter et al., 2022) and facilitating host virulence in plants (Partida-Martinez and Hertweck, 2005). Unfortunately, facilitating host virulence through toxin production in infected agricultural commodities may render them unsafe for human consumption (Steyn et al., 1983; Partida-Martinez and Hertweck, 2005). Upon infection, opportunistic fungal pathogens and plant/fruit endophytes interact closely and come to share the same ecological niche. Such interactions often impose compositional shifts in the endophytic community (Rojas et al., 2020) but their influence on the pattern of endophytic fungal secondary metabolite production is largely unknown. Thus, the current study endeavored to assess and compare the overall endophytic fungal composition of naturally spoiled and *R. microsporus*-infected fruits, using tomatoes (*Solanum lycopersicum* L.) as a model.

Tomatoes are a globally significant commercial vegetable crop valued for their high nutritional, anticancer and antioxidant properties (Agarwal and Rao, 2000; Story et al., 2010). However, fungal infections, including *Rhizopus* spp., greatly influence their productivity and yield (Kwon et al., 2001; Wokocha and Oparah, 2004). Despite the available literature on plant pathogens of tomatoes, there is limited information on the impact of *Rhizopus*-endosymbiotic bacteria relationship on the native fungal community dynamics during tomato invasion and disease establishment and on fungal secondary metabolite production in the tomatoes. Therefore, the study aimed at evaluating the magnitude of pathogen-induced changes in the native fungal community structure and determining the influence on fungal secondary metabolite production patterns.

## Materials and methods

### Fungal strains

*Rhizopus* was isolated by directly sprinkling 1 g of rhizosphere soil on malt yeast extract agar (MYA) prepared according to the manufacturer's instructions. The soil samples were collected from Witsand nature reserve, a desert ecoregion in the Northern Cape, South Africa. Inoculated plates were incubated for 3 days at 28°C. The same medium was used to maintain the pure cultures for further analysis. The isolates were identified after 28S rRNA gene sequencing as two strains of *R. microsporus* as described in the subsequent section.

### Molecular identification of fungal isolates

The pure cultures of the *Rhizopus* isolates were used for whole genomic DNA extraction using the Quick-DNA^TM^ Fungal/Bacterial Miniprep Kit (Zymo Research, Thermo Scientific, SA) according to the manufacturer's protocol with the following modifications: (1) instead of 5 min, the mycelial tissues were vortexed for 30 min and (2) 100 μL of elution buffer was replaced with 70 μL. The quality of the eluded DNA products was confirmed using the NanoDrop 1000 spectrophotometer (Thermo Scientific, SA). The resultant DNA extracts were subjected to PCR amplification of the fungal large ribosomal subunit (LSU) 28S rRNA gene using 25 μL PCR mixtures. Each PCR mixture for the fungi consisted of 12.5 μL of 2x DreamTaq PCR Master mix (Qiagen), 1 μL each of the forward LROR (5′- ACCCGCTGAACTTAAGC-3′) and reverse LR7 (5′- TACTACCACCAAGATCT-3′) primers (Integrated DNA Technologies, SA) at 10 nM concentrations, 1 μL of template DNA, and 9.5 μL of PCR Nuclease-free water (Qiagen). The selected primers are considered suitable for the taxonomic identification of fungi from early divergent lineages, targeting the variable D1–D4 domains of the LSU gene region (Tedersoo et al., 2015). The PCR conditions were as follows: 2 min at 95°C for initial denaturation, 35 cycles of 30 s at 95°C, 30 s at 61°C, 45 s at 72°C for annealing, and 10 min at 72°C for final elongation. The resultant PCR amplicons were analyzed by 1% (v/v) agarose TAE-gel electrophoresis and visualized using GelDoc Go Imaging System, BioRad. Successfully amplified PCR products were submitted for Sanger sequencing at Inqaba (Inqaba Biotec^TM^, SA) using the same primers as those for PCR. The obtained fungal 28S rRNA forward and reverse sequences were assembled and edited using Geneious Prime v.2024.0.7 (https://www.geneious.com) to generate contiguous sequences. The obtained contiguous sequences were used as queries in BLASTn searches of the National Center for Biotechnology Information (NCBI). The initial DNA samples were then used to detect putative endobacteria as described below.

### Molecular detection of endobacteria

Following the identification of *Rhizopus microsporus*, the initial gDNA samples were further subjected to PCR amplification using bacterial 27F (5′-AGAGTTTGATCCTGGCTCAG-3′) and 1492R (5′-GGTTACCTTGTTACGACTT-3′) rRNA gene primers (Inqaba Biotec^TM^, SA) to detect putative endobacteria in fungal DNA extracts according to Okrasińska et al. (2021). The 25 μL PCR volume consisted of 12.5 μL DreamTaq PCR master mix (Qiagen), 1 μL each of the forward and reverse primers at 10 nM concentrations, 1 μL template DNA, and 9.5 μL PCR Nuclease-free water (Qiagen). The PCR conditions were as follows: 2 min at 95°C for initial denaturation, 35 cycles of 30 s at 95°C, 30 s at 61°C, 45 s at 72°C for annealing, and 10 min at 72°C for final elongation. PCR success was verified by 1% (v/v) agarose TAE-gel electrophoresis. Sanger sequencing of PCR amplicons was conducted at Inqaba (Inqaba Biotec^TM^, SA) using the same primers as those for PCR amplification. The resultant raw 16S rRNA gene forward and reverse sequences were assembled and edited using the Geneious Prime v.2024.0.7 algorithm to generate contiguous sequences. Both fungal and bacterial sequences were then used for phylogenetic inference.

### Phylogenetic reconstructions for fungal and bacterial sequences

Fungal and bacterial sequences, separately, were edited in Mesquite v.3.81 (Maddison and Maddison, 2023) and aligned using the MUSCLE algorithm (Edgar, 2004). Best-fit nucleotide substitution models were identified, and maximum likelihood phylogenetic reconstructions were performed in the RAxML-GUI v.2.0 graphical interface (Edler et al., 2021). Maximum-likelihood phylogenetic trees were inferred using the generalized time reversible (GTR) and inverted gamma-distributed rate variation model with 1,000 bootstrap replicates. The obtained trees were edited using the interactive Tree of Life (iTOL) online platform (Letunic and Bork, 2021). Following the phylogenetic assignments, we sought to obtain aposymbiotic lines of the two *R. microsporus* strains for further analysis.

### Symbiont-free *R. microsporus* strains

To obtain *R. microsporus* without symbiotic bacteria, the isolated pure cultures were subjected to alternate cultivation in liquid and in solid MYA medium containing ciprofloxacin (20 μg/ml) and tetracycline (15 μg/ml) following a slightly modified method described by Partida-Martinez et al. (2007a). The procedure was repeated five times until aposymbiotic fungal strains were obtained. The elimination of endobacteria was monitored and confirmed through 16S rRNA PCR amplification. Once confirmed, both aposymbiotic and symbiotic *R. microsporus* strains were used to prepare the inoculum for pathogenicity testing.

### *R. microsporus* inoculum preparation

To prepare the inoculum, *R. microsporus* cultures were inoculated on Potato Dextrose Agar (PDA) for 3 days at 28°C. Following the methods described by Qin et al. (2022), the petri dishes containing freshly grown mycelia were then flooded with sterile distilled water and the mycelia scraped and collected into falcon tubes. Using a spectrophotometer, the concentrations of the fungal suspensions were determined and adjusted with sterile distilled water to 4.48 × 10^7^ CFU/mL and used for pathogenicity testing in tomato fruits accordingly.

### Pathogenicity test

Healthy, firm tomato fruits with no apparent diseased spots were purchased from a local supermarket in Potchefstroom, North-West province, South Africa, and kept at 4°C for further analysis. At the beginning of the inoculation experiment, the fruits were surface sterilized with freshly prepared 70% ethanol, thoroughly rinsed in sterile distilled water, and left to dry on individual sterile plastic bags before wounding. The tomato inoculation procedure was adapted from Qing and Shiping (2000). Using the tip of a sterile 20 μL filter pipette, uniform 10 mm deep wounds were made at the bottom side (as when hanging on the plant) of the fruit. A 10 μL suspension of the following treatments was made into each wound: (A) 4.48 × 10^7^ CFU/mL of endobacteria-positive *R. microsporus* suspension and (B) 4.48 × 10^7^ CFU/mL of aposymbiotic *R. microsporus* mycelial suspension. The treatment labeled as (C) was uninoculated wounded fruits. The fruits were then placed in individual sterile polyethene zip-lock bags to retain humidity and incubated for 7 days at ambient temperature to simulate shelf-life storage conditions. The relative humidity and temperature were maintained between 50 and 60% and 25–27°C for the incubation period, respectively, as previously suggested for appropriate storage of tomato fruits (Lamidi et al., 2020). The percentage spoilage incidence was determined using an adapted formula by Kabiru and Yusuf (2024) as follows:


$$\begin{array}{l} \text{\% spoilage incidence}\\ =\;\frac{Number\;of\;infected\;tomato\;fruits}{Total\;number\;of\;tomato\;fruits\;in\;the\;treatment}\;X\;100 \end{array}$$

The diameters of the spot inoculations were recorded every 24 h throughout the incubation period. There were six replications for each treatment. To confirm the presence of the inoculated *R. microsporus* strain, the spoiled tomatoes were homogenized using a sterile pestle and mortar following the termination of the experiment after the 7-day incubation period. The homogenate was serially diluted and pour-plated on PDA—a nutrient-rich medium previously used to isolate filamentous fungi from spoiled tomato fruits (Mugao and Birgen, 2021). The plates were incubated at 28°C for 3 days. The isolates of *R. microsporus* were sub-cultured based on morphological observations. The identities of the isolated strains were confirmed through PCR amplification and Sanger sequencing of the 28S rRNA gene, following the same conditions as previously described. To evaluate the influence of *R. microsporus* infections on the native endophytic fungal communities, the homogenates were analyzed as described below.

### Metabarcoding analysis of fungal endophyte communities

The homogenates were used for total genomic DNA extraction using the Quick-DNA^TM^ Bacterial/Fungal Miniprep kit (ZYMO research) according to the manufacturer's instructions with a few modifications. Instead of 100 μL elution buffer, only 70 μL was used. The obtained DNA was quantified using a NanoDrop spectrophotometer (NanoDrop One, Thermo Scientific, SA). The extracted DNA was submitted for fungal library preparation and Illumina NovaSeq at Novogene (Novogene Co., Singapore) and the ITS1 rRNA gene region was amplified with specific primers; ITS1F (CTTGGTCATTTAGAGGAAGTAA) and ITS2 (GCTGCGTTCTTCATCGATGC), and barcodes (Novogene Co., Singapore). The PCR mixtures contained 15 μL of Phusion^TM^ High-Fidelity PCR Master Mix (New England Biolabs), 0.2 μM of each primer and 10 ng target DNA. The cycling conditions were as follows: 98°C for 1 min, 30 cycles at 98°C for 10 s, 50°C for 30 s, 72°C for 30 s, and final extension at 72°C for 5 min. Successful PCR products were verified on 2% (v/v) agarose gel electrophoresis and mixed PCR products were purified using the Qiagen Gel Extraction Kit (Qiagen, Germany). Sequencing libraries were generated using the NEBNext^TM^ Ultra^TM^ IIDNA Library Prep Kit (Cat No. E7645) according to the manufacturer's recommendations. The quality of the library was evaluated on the Qubit@ 2.0 Fluorometer (Thermo Scientific) and Agilent Bioanalyzer 2100 system. Sequencing was performed on an Illumina NovaSeq platform, generating 250 bp paired-end reads. Sequenced reads were demultiplexed and assessed for low-quality reads using FastQC, v0.12.1 (Andrews, 2010). The adapter sequence and low-quality regions were trimmed off using Trimmomatic v0.38 (Bolger et al., 2014) and the reads with average nucleotide bases quality score of less than 15 (Phred Q score) were eliminated. The sequenced reads were then clustered into amplicon sequence variants (ASVs) using the DADA2 denoiser (Callahan et al., 2016) with default parameters in the Quantitative Insight into Microbial Ecology (QIIME2, v2024.2) software (Bolyen et al., 2019). The denoised reads were then classified using a trained classifier (ITS1–ITS2 region) of the UNITE v9.0 v25.07.2023 reference database (Abarenkov et al., 2024). ASVs assigned to chloroplasts (at order taxonomic level) and mitochondria (at family taxonomic level) were eliminated. Additionally, the ASV count table was then rarefied to an even depth across samples before computing alpha-diversity metrics. Then, the previously homogenized samples were also used to evaluate the influence of *R. microsporus* infections on the pattern of secondary metabolite production by fungal endophytes.

### Fungal secondary metabolite analysis of tomatoes

Tomatoes were homogenized using a sterile mortar and pestle, with the equipment sterilized with freshly prepared 70% ethanol between each fruit. Analysis of tomatoes for fungal secondary metabolites was performed according to the protocol of Sulyok et al. (2024). Five grams of the homogenized tomatoes were placed in sterile falcon tubes at ambient temperature, 20 mL extraction solvent (acetonitrile/water/acetic acid 20:79:1, v/v/v) was added, and the suspension was shaken for 90 min using a rotary shaker (GFL 3017, GFL; Burgwedel, Germany). Five hundred microlitres (μl) of the supernatants were transferred into HPLC vials and diluted with 500 μl of dilution solvent. After appropriate mixing, 5 μL of the diluted extracts were injected into the LC–MS/MS system without further pre-treatment. Analysis was done using a QTrap 5500 MS/MS system (Sciex, Framingham, MA, USA) coupled to a 1290 series UHPLC system (Agilent Technologies, Waldbronn, Germany). Chromatographic separation was obtained at 25°C on a Gemini C_18_-column, 150 × 4.6 mm i.d., particle size 5 μm, equipped with a C_18_ security guard cartridge, 4 × 3 mm i.d. (both Phenomenex, Torrance, CA, USA). For elution, a binary methanol/water gradient containing 1% acetic acid and 5 mM ammonium acetate was applied with a flow rate of 1 ml/min. The injection volume was 5 μl. ESI-MS/MS data was acquired in the scheduled multiple reaction monitoring (sMRM) mode both in positive and negative polarity in two separate chromatographic runs.

### Statistical analysis

The results obtained from the pathogenicity tests were analyzed using GraphPad Prism 10.4.0 (GraphPad software, Boston, Massachusetts, USA). Briefly, the measurements obtained for the lesion diameters were averaged and expressed as standard deviations and used to plot a line graph. The averages and standard deviations were calculated from all six treatment replications for all 7 days during incubation. Statistical analysis was performed using the one-way analysis of variance (ANOVA) and the Student *t*-test in Microsoft Office Excel 2010 to determine the statistical significance between treatments (*p* ≤ 0.05). Because treatments W2-50 aposymbiotic, W2-51 symbiotic, and W2-51 aposymbiotic could not yield any result for the lesion diameters (i.e., 0 mm), the *post-hoc t*-test between these treatments could not give any results and were not considered statistically significant.

### GenBank accession numbers

The nucleotide sequences generated in this study have been submitted in the NCBI database under GenBank accession numbers PP380448 (W2-50) and PP380449 (W2-51) for *R. microsporus*, and PP958808 (symbiont W2-50) and PP958809 (symbiont W2-51) for associated endobacteria.

## Results

### *R. microsporus* and *M. endofungorum* phylogenetic reconstructions

**Figure 1:**
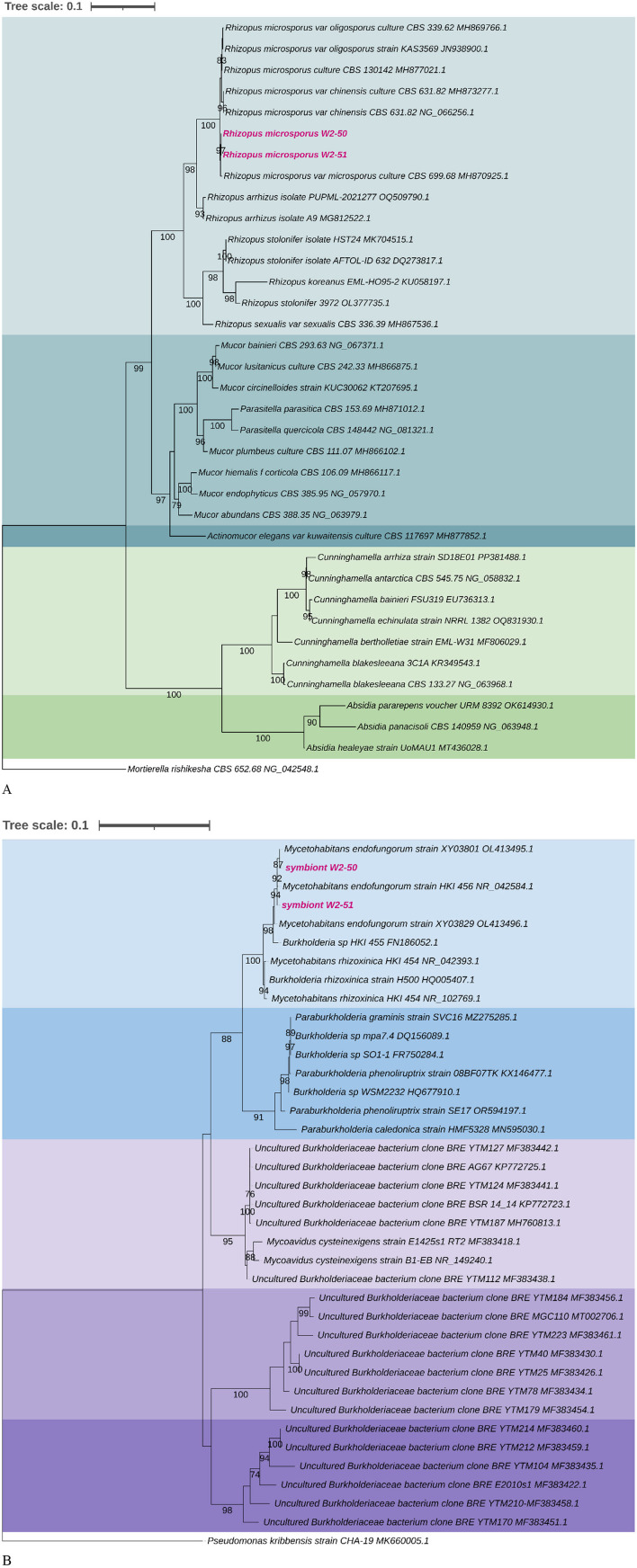
Maximum-likelihood phylogenetic trees based on the 28S **(A)** and 16S **(B)** rRNA gene sequences of *R. microsporus* isolates and *M. endofungorum* with selected closely related species from NCBI BLASTn searches. The 1,000 bootstrap replicates were calculated using RAx-ML-GUI. *Mortierella rishikesha* strain CBS 652.68 (NG042548) and *Pseudomonas kribbensis* strain CHA-19 (MK660005) were used as out groups for the 28S and 16S phylogenetic trees, respectively. The sequences of the strains used in this study are highlighted in bold pink.

Two fungal isolates obtained from desert soil were investigated for their potential to cause tomato rot. The 28S rRNA gene phylogenetic reconstructions proved the strains to be closely affiliated with *R. microsporus*. Successful amplification of their associated 16S rRNA gene sequences resulted in sequence lengths of ~1.5 kb that clustered closely with the *Mycetohabitans* species. Amplification of 16S rRNA genes in the fungal host proved that each is associated with a single bacterial strain. Among the two known toxin-producing *Mycetohabitans* symbionts of *R. microsporus*, the sequences generated in this study clustered with statistical support with *Mycetohabitans endofungorum*, specifically strain XY03801 (OL413495) and HKI 456^T^ (NR042584) for symbiont W2-50 and W2-51, respectively (Figure 1).

### Symbiont-free *R. microsporus* strains

Subjecting *R. microsporus* strains to antibiotics treatment allowed for the generation of pure cultures free of bacterial endosymbionts.

**Figure 2:**
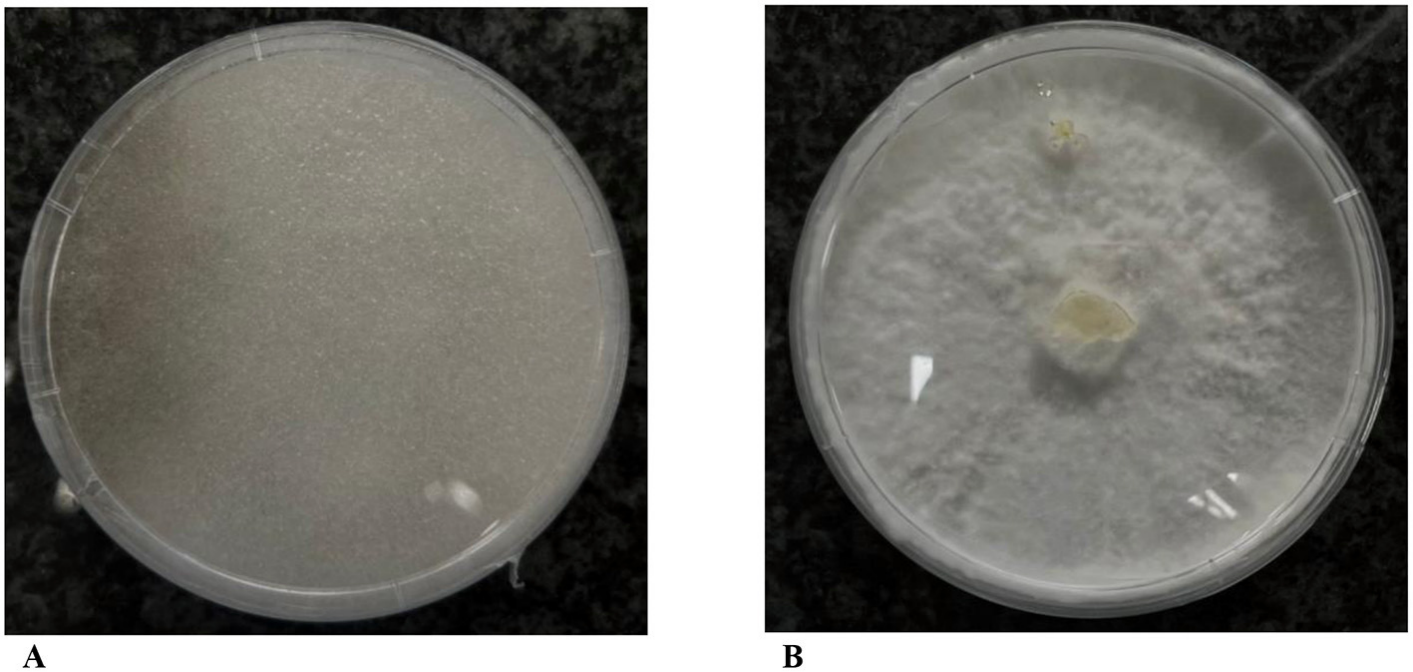
Effects of treating *R. microsporus* harboring endobacteria with ciprofloxacin and tetracycline. **(A)** Symbiotic *R. microsporus* W2-50 with spores. **(B)** Non-sporulating *R. microsporus* W2-50 after ciprofloxacin and tetracycline treatment.

Figure 2 compares the symbiotic and aposymbiotic *R. microsporus* strain W2-50. Notably, curing *R. microsporus* from their endosymbionts eliminates their sporulation ability, thus producing an endosymbiont-free strain with bulky hyphae.

### Pathogenicity testing

**Figure 3:**
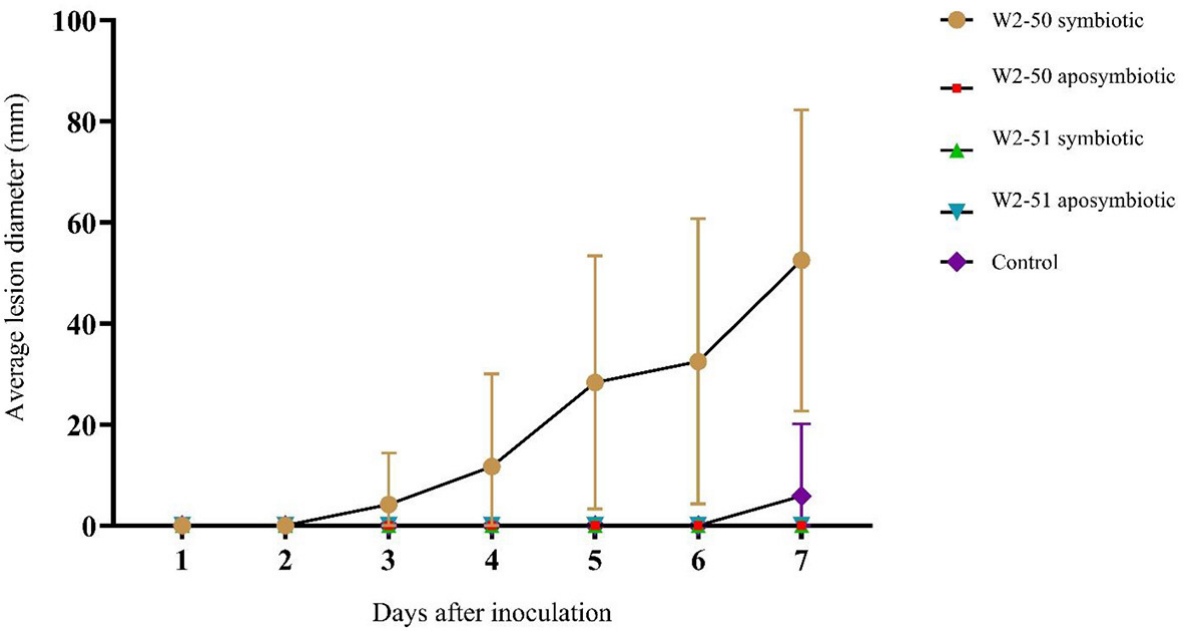
The comparison of the average lesion diameter between treatments post-inoculation. The statistical significance was determined using one-way ANOVA and *post-hoc* Student *t*-Test on microsoft office excel 2010 (*p* ≤ 0.05).

The spoilage incidence of non-inoculated (control) and inoculated (treated) tomatoes was evaluated by noting apparent spoilage following the incubation period (Table 1) and recording the lesion diameter of the spot inoculation for the duration of the experiment. There was significant spoilage across all treatments (*p* = 0.0014). As shown in Figure 3, the tomatoes treated with the symbiotic W2-50 strain caused more disease (4.167 ± 10.206–52.500 ± 29.791, *p* = 0.0307) than the other treatments. Although there was slight spoilage observed in the control group at day 7 (Figure 3), the *post-hoc* analysis showed that this was not statistically significant (*p* = 0.3370). Additionally, following the principle of Koch's postulate for pathogenicity testing, the symbiotic W2-50 strain could be successfully re-isolated and identified as the initial strain used to prepare the inoculum, thus proving its identity as the causative agent for the observed spoilage. In contrast, none of the strains from the other treatments could be re-isolated successfully. The inability to reisolate the aposymbiotic strain for the W2-50 treatment served as the first indication that the aposymbiotic inoculum may have become non-viable.

**Table 1:** Incidence (%) of tomato spoilage following *R. microsporus* inoculation (*n* = 6).

**Treatment**	**Spoilage incidence (*n* = 6)**	**Incidence (%)**
Wounded, uninoculated control	1/6	17% spoilage
Inoculated with symbiotic W2-50	4/6	67% spoilage
Inoculated with aposymbiotic W2-50	0	No spoilage
Inoculated with symbiotic W2-51	0	No spoilage
Inoculated with aposymbiotic W2-51	0	No spoilage

### ITS rRNA metabarcoding

**Figure 4:**
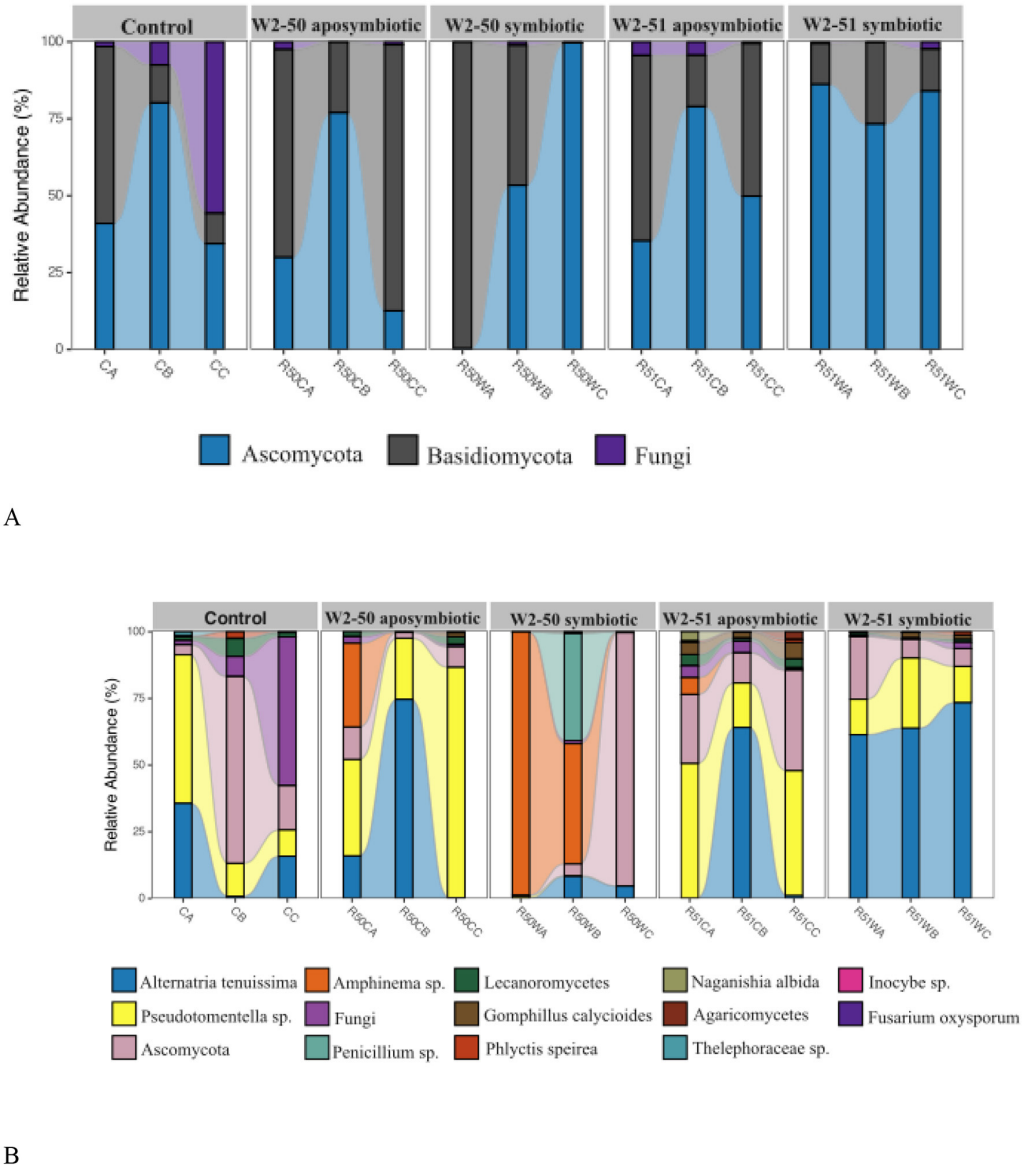
Fungal taxa distribution in tomato microbiota. Relative abundance of predominant fungi at phylum **(A)** and species **(B)** levels across endobacterial profile, as obtained by ITS rRNA gene metabarcoding of tomato treatments, including controls. C, aposymbiotic; W, symbiotic.

Metabarcoding of the ITS1/ITS2 region using total DNA samples from all experimental groups, including the controls, resulted in 1541533 reads. The total reads clustered into 583 ASVs. The phylum *Ascomycota* was the most prevalent across all the tomato samples, followed by the *Basidiomycota* phylum. From *Ascomycota*, the genus *Alternaria* was the most prevalent, whereas *Pseudotomentella* was prevalent among *Basidiomycota*. The most abundant fungal species were *Alternaria tenuissima, Amphinema* sp., *Thelephoraceae* sp., *Pseudotomentella* sp., and another unknown *Ascomycota* sp. (Figure 4).

Even though the tomato microbiome was dominated by *Basidiomycota* and *Ascomycota* phyla before (control) and after infection in all treatments, the relative abundance of *Basidiomycota* fungi was lower in tomatoes treated with the symbiotic W2-50 isolate. Instead, the relative abundance of *Ascomycota* increased, including species such as *Amphinema* and *Penicillium* sp. The increase in the abundance of these taxa was associated with a decrease in the taxa dominating the control treatment such as *A. tenuissima, Pseudotomentella* sp., *Lecanoromycetes* sp., and another unknown fungal species. On the other hand, the increase in the relative abundance of *A. tenuissima, Pseudotomentella* sp., *Lecanoromycetes* sp., and another unknown fungal species in the aposymbiotic W2-50 and the W2-51 (both aposymbiotic and symbiotic) was associated with the presence of another species, *G. calycioides*. Further evaluation of the fungal diversity within the tomato samples was done by measuring the microbial diversity within individual samples (Alpha-diversity) and quantifying the diversity between samples (Beta-diversity).

### Alpha-diversity indices

**Figure 5:**
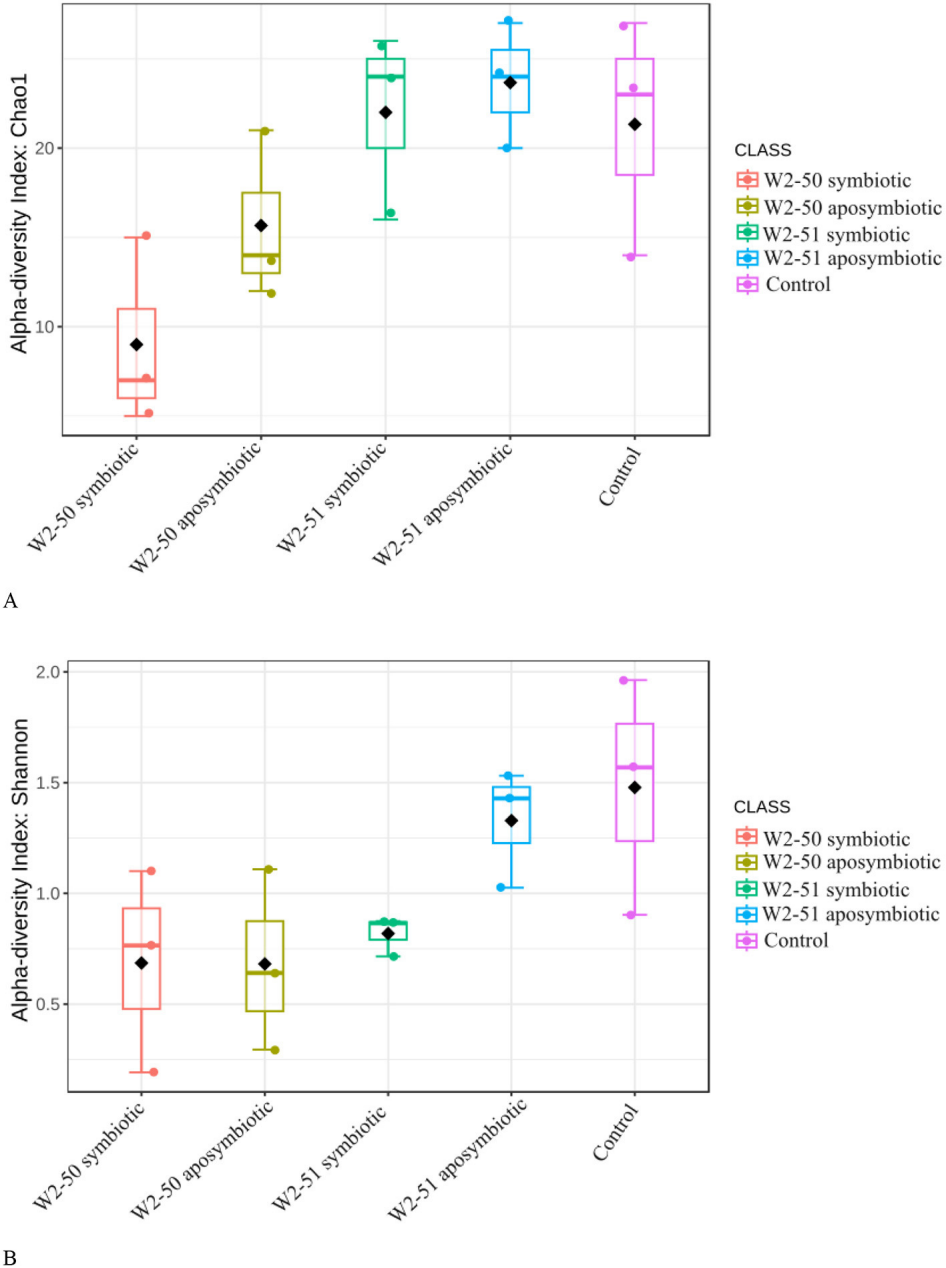
Comparison of alpha-diversity indices (**A**: Chao1, **B**: Shannon) between the microbiota of treated tomato fruits.

When considering the Chao1 Alpha diversity index—which provides an estimated total number of species in a community—(Figure 5A), the overall highest and lowest richness and abundance for endophytic fungal species across the tomato samples were observed in the aposymbiotic W2-51 and the symbiotic W2-50 treatments, respectively, highlighting potential out-competition of other fungal taxa upon infection by the symbiotic W2-50 isolate. Additionally, a noticeable increase in Chao1 Alpha diversity was observed in the aposymbiotic compared to the symbiotic treatments for both isolates, showing that the two lines behave completely differently upon infection into the fruits. When considering the Shannon Alpha diversity index—encompassing both species richness and evenness—(Figure 5B), the control samples revealed the highest diversity within the sample and across all the other samples, while the symbiotic W2-51 revealed the lowest diversity within the sample. Although the diversity within the sample was low, the overall diversity observed in the symbiotic W2-51 sample remained higher compared to both aposymbiotic and symbiotic W2-50 treatments. Therefore, although the samples treated with the W2-50 isolate showed higher individual diversity, they still had the lowest diversity across all samples.

### Beta-diversity index

**Figure 6:**
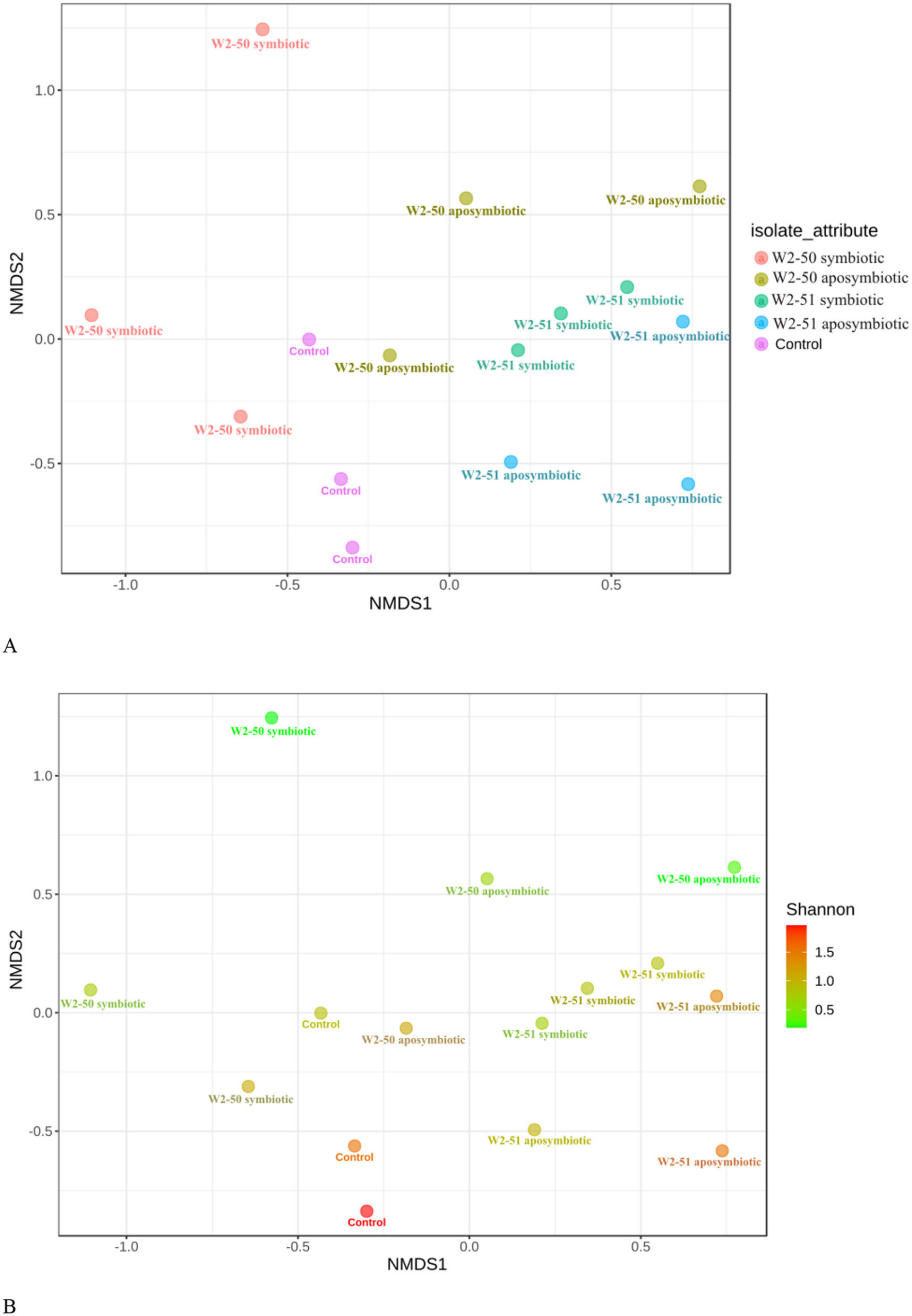
NMDS plot of Bray-Curtis dissimilarity distances for beta-diversity during *R. microsporus* infection of tomatoes. Each color represents the samples **(A)** and the Shannon *p*-value **(B)**.

A Non-metric MultiDimensional Scaling (NMDS) analysis, based on Bray-Curtis' dissimilarity of beta-diversity revealed symbiotic W2-51 and the control treatments as the main drivers of the diversity in the samples. This was shown by the independent clustering of these specific treatments from the other ones. Those samples that clustered farther apart (W2-50 symbiotic, W2-50 aposymbiotic, and W2-51 aposymbiotic) represented dissimilar microbiota between replicates of the same treatment (Figure 6A). The *p*-value for the Shannon index further revealed a significant difference in the microbiota between the replicates from the symbiotic W2-50 and the aposymbiotic W2-50 treatments (*p* < 1.0), separately. No significant difference was observed for the aposymbiotic and symbiotic W2-51 treatments. Furthermore, no significant difference was observed for the samples in the control group (Figure 6B). These results are consistent with the results observed for the relative abundance and diversity in the symbiotic W2-50 treatment, showing the highest individual diversity possibly due to increased abundance of *Amphinema* and *Penicillium* sp. compared to the other treatments, further highlighting the specific influence of this isolate on the fungal community of the tomatoes.

### Fungal secondary metabolites in tomatoes during spoilage

The treated tomato samples were analyzed for 900 different fungal metabolites, however, as seen in Table 2, only eight of the metabolites were detected across the samples. Three of the secondary metabolites are known to be produced by *Alternaria*, one by *Aspergillus* spp., whereas for the others multiple sources exist.

**Table 2:** LC-MS/MS analysis profiles for secondary metabolites detected in tomatoes treated with symbiotic *R. microsporus*, aposymbiotic *R. microsporus*, and uninoculated experimental groups.

**Sample ID**	**Alternariol**	**Alternariol methyl ether**	**Asperglaucide**	**Cyclo(L-Pro-L-Tyr)**	**Cyclo(L-Pro-L-Val)**	**Kojic acid**	**Tenuazonic acid**	**Tryptophol**
LOD (μg/kg)	0.072	0.029	0.06	0.9	0.45	7.5	2.07	1.2
Control	34.7	17.0	1.62	16.5	8.83	13,400	4.3	1,000
W2-50 Aposymbiotic	4.8	7.243	^*^	32.8	20.9	234	^*^	1,090
W2-50 symbiotic	^*^	0.52	0.373	^*^	5.7	^*^	^*^	547
W2-51 Aposymbiotic	^*^	1.14	^*^	18.7	6.13	^*^	^*^	863
W2-51 Symbiotic	^*^	^*^	0.143	28.5	9.93	^*^	^*^	410
Producer	*Alternaria*	*Alternaria*	Unspecific	Unspecific	Unspecific	*Aspergillus*	*Alternaria*	Unspecific

^*^ < LOD.

LOD represents the level of detection in μg/kg.

### *Alternaria* toxins

Alternariol (AOH), alternariol methyl ether (AME), and tenuazonic acid (TeA) are known metabolites produced by *Alternaria* species in spoiled foods. All three metabolites were detected in significantly higher levels of 34.7, 17.06, and 4.3 μg/kg in the control, respectively, compared to the other treatments. Surprisingly, the tomatoes inoculated with the symbiotic W2-50, showed the lowest AOH (< LOD), AME (0.52 μg/kg), and TeA (< LOD) compared to the control despite showing physical spoilage.

### *Aspergillus* metabolites

Only Kojic acid could be detected when considering the *Aspergillus* metabolites, with the control and the tomatoes inoculated with the aposymbiotic W2-50 showing the highest toxin levels (13,400 and 234 μg/kg, respectively).

### Unspecified metabolites

Several other metabolites were detected whose producer could not be specified. Higher levels of asperglaucide (1.62 μg/kg) and Tryptophol (1,000 μg/kg) were detected in the control tomatoes compared to all the other treatments, including those treated with the symbiotic W2-50 (0.373 and 547 μg/kg, respectively), highlighting a decrease in their production upon symbiotic W2-50 inoculation. However, tryptophol production was not significantly influenced in the aposymbiotic W2-50 treatment (1090 μg/kg) compared to the control. If anything, it was slightly increased. Another interesting observation was the significantly higher levels of asperglaucide and lower levels of tryptophol in the symbiotics compared to the aposymbiotic treatments.

In addition to the asperglaucide and tryptophol, two cyclic dipeptides; cyclo(L-Pro-L-Tyr) and cyclo(L-Pro-L-Val); were also detected, with the highest levels detected in the aposymbiotic W2-50 treatment (32.8 and 20.9 μg/kg, respectively). Contrarily, the lowest levels were detected in the symbiotic W2-50 treatment at < LOD and 5.7 μg/kg, respectively. This is especially interesting since the symbiotic W2-50 treatment showed the most significant level of spoilage.

## Discussion

The current study aimed to evaluate the magnitude of changes induced by a post-harvest fruit spoilage agent on the native fungal communities of tomatoes and the influence on secondary metabolite production by native endophytic fungi. *Rhizopus* spp. are among the most common fungi causing tomato infections (Sheikh et al., 2021). However, previous research has not attempted to investigate the effects of *Rhizopus microsporus* harboring endobacteria on the contamination of tomatoes (Wokocha and Oparah, 2004). Such research is particularly important since the prevalence of *Rhizopus* spp. in tomatoes purchased from supermarkets is relatively high (Sheikh et al., 2021; Kabiru and Yusuf, 2024). The results shed light on the effects of *R. microsporus* harboring endobacteria on tomato endophytic fungal communities and their secondary metabolite-producing patterns.

### Symbiont-free *R. microsporus*

Successful curing of *R. microsporus* strains isolated from nature using ciprofloxacin and tetracycline was supported by the loss of sporulation after five successive antibiotic treatments (Figure 2). These results are consistent with previous research highlighting the complete cessation of asexual reproduction in *R. microsporus* upon successful endobacterial removal. Previous studies have shown that the loss of endosymbionts is accompanied by loss of viability and the inability of the hosts to produce toxic rhizoxin and rhizonin metabolites, thus proving that the host relied on their bacterial symbiont for survival and toxin production (Partida-Martinez and Hertweck, 2005; Partida-Martinez et al., 2007a,b; Mondo et al., 2017). To evaluate the involvement of the associated endobacteria on the virulence of their fungal hosts, we investigated the pathogenicity of the symbiotic and aposymbiotic fungal strains on fresh tomato fruits.

### The establishment of *R. microsporus* W2-50 harboring endobacteria causes tomato spoilage

The symbiotic *R. microsporus* W2-50 strain successfully established itself within the inoculated tomato fruits, with observable whitish-gray mycelia with black sporangia growing from inside and around the fruits, characteristic of *Rhizopus* infections in fruits (Baggio et al., 2016). This treatment also demonstrated the highest percentage incidence (Table 1) and average lesion diameter (Figure 3) compared to the other treatments. Although there was significant spoilage across the treatments in the tomatoes (*p* = 0.0014), this was largely caused by inoculation with the symbiotic W2-50 strain (*p* = 0.0307) according to the *post-hoc* test. Contrastingly, none of the other treatments, including the aposymbiotic strain and the other symbiotic W2-51 strain elicited any significant spoilage to the inoculated fruits (*p* = 0.3370) (Figure 3), thus highlighting the strain-specific impact of the symbiotic W2-50 strain. To further validate the role of the symbiotic W2-50 isolate as the causative agent for the observed spoilage, this study sought to re-isolate the inoculated strains from the fruits upon cessation of the experiment per Koch's postulate, as previously suggested (Kabiru and Yusuf, 2024). Only the symbiotic *R. microsporus* W2-50 could be successfully re-isolated and identified as the original isolate inoculated into the fruits, thus highlighting its ability to successfully establish itself upon inoculation. Attempted re-isolation of the other fungal strains was unsuccessful, therefore indicating their inability to colonize and establish themselves within the fruits possibly due to loss of viability. Successive PCR amplification and Sanger sequencing of the fungal 28S and its associated bacterial 16S rRNA genes from the genomic DNA samples extracted from the symbiotic W2-50 inoculum proved its identity as *R. microsporus* and its associated endosymbiont as *M. endofungorum*. This served as further evidence that the inoculated symbiotic *R. microsporus* W2-50 facilitated the observed spoilage. Indeed, the involvement of fungi (e.g., *Fusarium, Alternaria*, and *Rhizopus* spp.) as causative agents of tomato spoilage has been previously supported by successful re-isolation and identification of the responsible species (Lemma et al., 2014; Mugao and Birgen, 2021; Sheikh et al., 2021).

### Endobacterium of symbiotic *R. microsporus* W2-50 may facilitate host virulence through toxin production

Inoculation of tomato fruits with the same strain without endobacteria (aposymbiotic W2-50) could not cause any apparent spoilage in the fruits, thus serving as an indication of its non-viable nature and the potential role of the associated endosymbiont W2-50 in host virulence. The inability of the aposymbiotic strain to establish itself within the fruits can be attributed to its loss of sporulation ability and thus limited reproductive success upon endosymbiont removal as documented in phylogenetically related strains (Partida-Martinez et al., 2007a; Mondo et al., 2017). Indeed, the symbionts detected in this study clustered closely with previously identified rhizonin- and rhizoxin-producing *M. endofungorum* strain HKI 465^T^ (Jennessen et al., 2005; Partida-Martinez et al., 2007a,b) known to control the sporulation and virulence of its *R. microsporus* CBS 112285 host (Partida-Martinez et al., 2007a; Mondo et al., 2017). Therefore, we hypothesize that the symbiont W2-50 may possess similar toxic traits that facilitate host virulence in tomatoes since it appeared to belong to the same taxonomic clade of toxin-producing strains (Figure 4).

### Symbiotic *R. microsporus* W2-50 infection alters community structure to favor spoilage-causing fungal communities

Previous culture-dependent identification of fungal communities responsible for tomato spoilage revealed filamentous fungi such as *Fusarium* spp., *Aspergillus* spp., *Penicillium* spp., *Alternaria* spp., *Mucor* spp., and *Rhizopus* spp. as the most prevalent fungi contributing to the spoilage (Bello et al., 2016; Sheikh et al., 2021; Kabiru and Yusuf, 2024). Because this method may be biased against uncultivable communities and offer a limited representation of yeasts of the *Basidiomycota* phylum (Rojas et al., 2020), our study sought to unravel the influence of *R. microsporus* infections on endophytic fungal communities in tomatoes through metabarcoding of the ITS1 gene region from tomato DNA extracts. Although our results could not indicate the presence of the inoculated *R. microsporus* strains through ITS metabarcoding, this can be attributed to the sequencing bias offered by ITS primers, favoring the amplification and sequencing of short-length sequences such as Ascomycota over longer sequences (Kauserud, 2023). Nevertheless, our results showed a shift in community structure from abundant *Alternaria tenuissima, Lecanoromycetes*, and *Pseudotomentella* sp. to *Amphinema* and *Penicillium* sp. in the symbiotic W2-50 treatment (Figure 4). Although the presence and role of *Amphinema* sp. in tomatoes is unknown, the involvement of *Penicillium* sp. alongside *Rhizopus* sp. in tomato spoilage is well-documented (Bello et al., 2016; Sheikh et al., 2021). Indeed, the tomatoes treated with the symbiotic W2-50 strains showed the highest level of *Penicillium* sp. and spoilage compared to the treatments with no apparent spoilage. Therefore, we presume that the presence of this fungal taxa may have facilitated the observed spoilage in this treatment. The other treatments, however, showed the presence of a new species *Gomphillus calycioides* (Phylum *Ascomycota*), whose role in tomatoes is also unknown. We speculate that the absence of this species in the symbiotic W2-50 treatment might be due to increased competitiveness of the symbiotic W2-50 isolate due to the possible possession of potent rhizoxin produced in symbiosis with the associated endobacteria. This claim is supported by the known role of rhizoxin in facilitating host toxicity against other fungi, including several *Ascomycota* and *Basidiomycota* species (Schmitt et al., 2008; Richter et al., 2022).

### *R. microsporus* infections influence the pattern of secondary metabolite incidence

To determine the influence of *R. microsporus* infections on secondary metabolite production by native fungal communities, LC-MS/MS analysis of the tomato fruits was carried out. Considering metabolite levels in the control and W2-50 treatments, the detected metabolites were generally higher in the control than in the symbiotic W2-50 treatment. Furthermore, all the metabolites except asperglaucide were higher in the aposymbiotic compared to the symbiotic W2-50 treatment, with a few metabolites even higher in the aposymbiotic than the control (Table 2). The decrease in the metabolite levels correlates with the reduced relative abundance of pre-existing taxa upon infection with the symbiotic W2-50 isolate, highlighting the potential out-competition of pre-established taxa by symbiotic *R. microsporus* W2-50 upon infection.

While AOH and AME are known metabolites of various *Alternaria* spp. (Awuchi et al., 2021), and Kojic acid a metabolite of *Aspergillus* spp., the true producers of cyclo(L-Pro-L-Tyr), cyclo(L-Pro-L-Val) and tryptophol could not be specified through LC-MS/MS analysis or ITS metabarcoding in this study (Figure 4). Previous research has indicated that in addition to *Bacillus* and *Streptomyces* spp., cyclo(L-Pro-L-Tyr) and cyclo(L-Pro-L-Val) could be produced by fungi such as *Aspergillus fumigatus* (Álvarez et al., 2023) and tryptophol by various fungal species (Palmieri and Petrini, 2019). However, despite the production of *Alternaria* metabolites being further supported by the detection of *Alternaria tenuissima* in all samples, neither *Aspergillus* spp. nor any of the fungal species specified by Palmieri and Petrini (2019) could be detected through ITS sequencing to support their potential identity as the true producers of kojic acid, tryptophol and cyclic dipeptides in the current study. Interestingly, the two cyclic dipeptides detected in this study [cyclo(L-Pro-L-Tyr) and cyclo(L-Pro-L-Val)] have been previously reported to possess antifungal properties against several filamentous fungi, including but not limited to *Aspergillus flavus, Penicillium expansum*, and *Fusarium oxysporum* (Mishra et al., 2022). Although this was not specifically reported for *Rhizopus* species, it is worth noting that the treatments without significant spoilage possessed higher levels of these secondary metabolites compared to the symbiotic W2-50 treatment that showed spoilage (Table 2), thus suggesting potential antimicrobial activity in these treatments. This is further supported by the absence or low relative abundance of *Aspergillus, Fusarium*, and *Penicillium* sp. in these treatments compared to the presence of *Penicillium* in the symbiotic W2-50 treatment (Figure 4B).

Previous research has reported higher AOH (12.2 μg/kg) and AME (13.5 μg/kg) levels in completely spoiled tomatoes, followed by the detection of their associated fungal producers (Van de Perre et al., 2014), whereas our results showed much higher levels of AOH (34.7 μg/kg) and AME (17.06 μg/kg) in the controls even though there was no observable spoilage in the controls. However, considering the treatments, much lower levels of AOH and AME were detected (Table 2). Correlating these results with the fungal taxa distribution, the tomatoes infected with symbiotic W2-50 had a reduction in *Alternaria* sp. abundance (Figure 4). Interestingly, not all treatments elicited observable spoilage, yet potentially toxic *Alternaria* secondary metabolites were detected, suggesting that physical spoilage does not necessarily reflect safety. *Alternaria* mycotoxins are among the regulated fungal toxins by the European Food Safety Authority (EFSA) due to their detrimental health effects on humans and animals when consumed at high concentrations. AOH and AME are considered among the most common and toxic mycotoxins to animals, eliciting cytotoxic and genotoxic effects (Escrivá et al., 2017). For this reason, the EFSA set the estimates for chronic dietary exposure for AOH to 1.9–39 (0.0019–0.039 μg/kg) and for AME to 0.8–4.7 (0.0008–0.0047 μg/kg) ng/kg b.w/day, while the Panel on Contaminants in food (CONTAM Panel) set the toxicological concern threshold (TTC) for these toxins to 2.5 (0.0025 μg/kg) ng/kg b.w/day (EFSA, 2011). Therefore, the levels of *Alternaria* metabolites detected in this study, especially in non-spoiled tomatoes, far exceed the set standards and regulations for food safety.

## Conclusion

The current study provides insight into tomato spoilage dynamics related to infection by *Rhizopus microsporus* and its endobacteria, including shifts in fungal endophyte community and associated secondary metabolites. While previous studies have reported the functional role of *Mycetohabitans endofungorum* in toxin production by the host fungus infecting foodstuff, not all *R. microsporus* isolated from contaminated commodities have been investigated for the presence to harbor endobacteria. Thus, our study highlights the need for comprehensive research into the potential presence of toxin-producing endobacteria in *R. microsporus* infecting foodstuff and other perishable commodities, their functional roles, as well as quality monitoring since their presence in *R. microsporus* may prove detrimental to consumers if neglected. The loss of sporulation in *R. microsporus* upon elimination of endobacteria with antibiotics has been previously linked to loss of host pathogenesis, thus raising the prospect of antibacterial treatment for the management of symbiotic *R. microsporus* infections.

## Data Availability

The original contributions presented in the study are publicly available. This data can be found here: https://www.ncbi.nlm.nih.gov/genbank, accession numbers PP380448 (W2-50) and PP380449 (W2-51) for *R. microsporus*, and PP958808 (symbiont W2-50) and PP958809 (symbiont W2-51) for associated endobacteria.
